# Protective effects of *Phyllanthus phillyreifolius* extracts against hydrogen peroxide induced oxidative stress in HEK293 cells

**DOI:** 10.1371/journal.pone.0207672

**Published:** 2018-11-16

**Authors:** Dovilė Grauzdytė, Audrius Pukalskas, Wildriss Viranaicken, Chaker El Kalamouni, Petras Rimantas Venskutonis

**Affiliations:** 1 Department of Food Science and Technology, Kaunas University of Technology, Kaunas, Lithuania; 2 Université de la Réunion, UM 134 Processus Infectieux en Milieu Insulaire Tropical (PIMIT), INSERM U1187, CNRS UMR9192, IRD UMR249, Plateforme Technologique CYROI, Sainte Clotilde, France; College of Agricultural Sciences, UNITED STATES

## Abstract

*Phyllanthus phillyreifolius*, a plant species indigenous to Reunion Island, is used in folk medicine for treating diarrhea and as a diuretic. In the present study acetone and hydroethanol extracts of *P*. *phillyreifolius* were evaluated for their cytotoxicity and antioxidant effects using *in vitro* (TPC, ABTS, DPPH, FRAP, ORAC) and *in cellulo* (MTT, DCFH-DA, RT-qPCR) assays. Major compounds were evaluated using UPLC–QTOF–MS. MTT cell viability assay showed low cytotoxicity of extracts towards human embryonic kidney 293 (HEK293) cell line. Both extracts were rich in polyphenols (mainly ellagitannins) and showed high antioxidant potential and intracellular ROS decreasing effect. Preconditioning of HEK293 cells with extracts influenced gene expression of antioxidant enzymes, however ROS level decreasing effect was more related to their capacity to scavenge free radicals and with their reducing power. Strong antioxidant activity of extracts as well as the presence of geraniin supports the use of *P*. *phillyreifolius* in traditional medicine.

## Introduction

The plants of genus *Phyllanthus* are widely distributed in tropical and subtropical countries and have been commonly used as traditional medicines for numerous ailments including diabetes, dysentery, tumors, hypertension, obesity, asthma, malaria, liver and kidney diseases [1]. Most of the above mentioned diseases are related to oxidative stress, caused by the excessive amount of reactive oxygen species (ROS), which may damage important to cell structures biomolecules like DNA, lipids and proteins [2]. Although the human body has endogenous defense system, in some cases this system may not be sufficient to keep the balance between the production and inactivation of ROS, and it is hypothesized that the bioactive dietary compounds of plants with antioxidant potential may play a definite role in modulating ROS level and thereby decrease the risk of these diseases [3].

Numerous studies have focused on search of new natural sources of antioxidant compounds. To identify antioxidant potential of plant extracts and pure compounds, in vitro assays are widely used, but they cannot accurately predict antioxidant behavior in living systems. For this reason cell-based assays are gaining importance as they provide a biological perspective [4]. Recent studies showed that plant extracts can exert cytoprotective role against oxidative stress induced by various oxidants by direct antioxidant activity and/or by regulating endogenous antioxidant enzymes levels and activities [5, 6, 7].

*Phyllanthus phillyreifolius*, a plant species native to Reunion Island, is locally known in folk medicine for treating diarrhea and as a diuretic [8]. Considering that oxidative stress is one of the etiologic factors involved in diarrhea the present study was designed to evaluate the protective action of polyphenol-rich extracts on HEK293 cells against H_2_O_2_ induced oxidative stress by measuring the production of ROS and gene expression of antioxidant enzymes. In addition, the cytoxicity of extracts was also evaluated on HEK293 cells. Finaly, total phenolic content and antioxidant properties of *P*. *phillyreifolius* were evaluated using *in vitro* assays, whereas phytochemical characterisation was performed using UPLC–QTOF–MS analysis.

## Materials and methods

### Chemicals and reagents

2,2-Diphenyl-1-picrylhydrazyl radical (DPPH^•^, 98%), 2,2-azino-bis(3-ethylbenzothiazoline-6-sulfonic acid) diammonium salt (ABTS, 98%), fluorescein (FL), 2,2-azobis(2-amidinopropane) dihydrochloride (AAPH), gallic acid, 6-hydroxy-2,5,7,8- tetramethylchroman-2-carboxylic acid (Trolox, 97%), 2,7-dichlorofluorescin diacetate, 3-(4,5- dimethylthiazol-2-yl)-2,5-diphenyltetrazolium bromide (MTT), trypan blue, hydrogen peroxide (H_2_O_2_) were purchased from Sigma-Aldrich (Steinheim, Germany). 2.0 M Folin-Ciocalteu phenol reagent, KCl, Na_2_HPO_4_, K_2_S_2_O_8,_ NaCl, Na_2_CO_3_ and HPLC grade acetonitrile were from Merck (Darmstadt, Germany); KH_2_PO_4_ from Jansen Chimica (Beerse, Belgium); 2,4,6-tripyridyl-s-triazine (TPTZ) from Fluka Chemicals (Steinheim, Switzerland). Dimethyl sulfoxide (DMSO) was from Sigma-Aldrich (Saint-Quentin-Fallavier, France); reference compounds, gallic acid, rutin and quercitrin hydrate from Sigma Chemical Co. (St. Louis, MO, USA), ellagic acid from Fluka Biochemica (Buchs, Switzerland), geraniin from ALB technology (Mongkok Kowloon, Hong Kong, China). Analytical grade solvents, hexane, acetone, methanol and acetic acid were from StanLab (Lublin, Poland); agricultural origin ethanol (96.6%) from Stumbras (Kaunas, Lithuania). HPLC grade solvents for chromatographic analyses were purchased from Sigma-Aldrich Chemie (Steinheim, Germany). Ultrapure water was produced using a Simplicity 185 system (Millipore, MA, USA).

### Plant material and preparation of extracts

The aerial parts of *Phyllanthus phillyreifolius* were collected in south west of Reunion Island in November 2013. The plants were dried overnight at 37°C. The voucher specimens (UR-PP2013/1) were deposited in the herbarium of the University of Reunion. Before extraction the plants were ground in a laboratory mill Retsch ZM200 (Retch GmbH, Haan, Germany) to 1 mm particle size. For acetone (AC) extract 12 g of plant material was placed into a cellulose thimble and extracted in a Soxhlet extractor (Behr Labor-Technik, Düsseldorf, Germany) at 56°C for 3 h. For hydroethanol (EH) extract 5 g of plant material was treated by stirring with 100 mL of ethanol:water (70:30, v/v) at room temperature for 1 h. The obtained extract was filtered over Whatman No.1 filter paper and the filtrate was collected. This procedure was repeated one more time. After extractions, acetone and ethanol were evaporated in a rotary vacuum evaporator (Büchi, Flawil, Switzerland) at 42°C while residual water was removed by freeze-drying. The yields of AC and EH extracts were 11.53±1.13 and 33.63±1.15%, respectively. Dry extracts were stored in a freezer prior further analysis.

### Cell culture

Human Embryonic Kidney 293 (HEK-293T) cells (ATCC CRL-1573, Manassas, VA, USA) were cultured at 37°C with 5% CO_2_ in DMEM medium (Gibco/Invitrogen, Carlsbad, CA, USA), supplemented with 10% heat inactivated fetal bovine serum (Dutscher, Brumath, France) and 2 mmol/L L-glutamine, 1 mmol/L sodium pyruvate, 100 U/mL penicillin, 0.1 mg/mL streptomycin and 0.5 μg/mL fungizone (PAN Biotech, Germany). Along the experiments, the cells were monitored by microscopic observation in order to detect any morphological changes.

### Assays in cells

#### Cytotoxicity assay

The cytotoxicity was evaluated by spectrophotometric MTT assay [9] with some modifications. Briefly, serial dilutions of extracts (1000 μg/mL stock) in DMEM were added to 1 x 10^4^ cells/well in a 96-well plate and incubated for 24 h. The supernatant was discarded, and a solution containing MTT (5 mg/mL) was applied for 1 h. After incubation, the supernatant was removed and the formazan crystals were solubilized with 100 μL of DMSO. Optical density (OD) was measured at 570 nm with a background subtraction at 690 nm. Data from three independent experiments were normalized using the following equation: cell viability (%) = (OD sample value)/(OD cell control) x 100. The concentration inhibiting the viability in 50% of cells (IC_50_) was obtained by performing nonlinear regression followed by the construction of a sigmoidal concentration-response curve (variable slope; Graphpad Prism 5; La Jolla, CA, USA).

#### Cellular antioxidant activity (CAA)

Intracellular formation of ROS was evaluated by using oxidation sensitive DCFH-DA probes [10]. Briefly, HEK293 cells were cultivated overnight in black 96-well microplates at 1×10^4^ cells per well. After treatment with various concentrations of extracts (31.25÷250 μg/mL) for 3 h or 24 h, cells were washed with 50 μL of PBS and incubated for 45 min with 100 μL of DCFH-DA (10 μmol/L) in dark. To assess antioxidant activity, supernatant was removed and the cells were incubated for 1 h with or without 20 μL of H_2_O_2_ (100 μmol/L). The fluorescence was measured on a plate reader BMG-Labtech (Offenburg, Germany), using 485 nm excitation and 530 nm emission wavelengths.

#### RNA isolation, reverse transcription and quantitative real time PCR (QPCR)

Total RNA was extracted from cells with RNeasy kit (Qiagen, Courtaboeuf, France) and reverse transcription was performed using 500 ng of total RNA. Quantificative PCR was performed on a ABI7500 Real-Time PCR System (Applied Biosystems, Life Technologies, Villebon-sur-Yvette, France). Briefly, 10 ng cDNA was amplified using 0.2 μM of each primer and 1X GoTaq Master Mix (Promega, Charbonnières-les-Bains, France). Data were normalized to the internal standard GAPDH. For each single-well amplification reaction, a threshold cycle (Ct) was calculated using the ABI7500 program (Applied Biosystems) in the exponential phase of amplification. Relative changes in gene expression were determined using the ΔΔCt method and reported relative to the control. The primers used in this study are listed in S1 Table.

### *In vitro* antioxidant activity

For the assessment of antioxidant activity the extracts were dissolved in methanol at a concentration of 10 mg/mL and further diluted to a final concentration, suitable for the selected assay. Not fully dissolved extracts were treated in the ultrasonic bath ASTRA-SON^TM^, model 9H (Heat Systems Ultrasonics, NY, USA) and filtered. Absorbances were measured with Spectronic Genesys 8 spectrophotometer (Thermo Spectronic, Rochester, NY) in semi-micro cuvets (Ratiolab GmbH, Dreieich, Germany). All experiments were carried out minimum in triplicates.

#### Total phenolic content (TPC)

TPC was determined spectrophotometrically according to the Folin–Ciocalteau’s method [11] with slight modifications. Firstly, commercial Folin-Ciocalteu’s reagent was diluted with distilled water at a ratio 1:9 (v/v). For analysis, 150 μL of extract solutions were mixed with 750 μL of Folin-Ciocalteu’s reagent and 600 μL of 7.5% sodium carbonate solution, left in dark for 2 hours and absorbance was measured at 760 nm. The TPC was calculated from the calibration curve using 150 μL gallic acid solutions (0–80 μg GA in mL ethanol) and the results were expressed in milligrams of GA equivalents per gram of extract (mg GAE/g_extract_).

#### DPPH^•^ scavenging assay

The assay was carried out by the method of Brand-Williams and colleagues [12] with some modifications. DPPH^•^ methanolic solution (~90 μmol/L, final absorption 0.800±0.03) was prepared daily before measurements. For analysis 500 μL of sample or MeOH (blank) were mixed with 1000 μL of DPPH^•^ solutions and left in dark for 2 hours. The decrease in absorbance value was measured at 517 nm. Radical scavenging capacity was calculated from the calibration curve using 500 μL Trolox solutions (0–50 μmol/L MeOH) and the results were expressed in μM of Trolox equivalents per gram of extract (μM TE/g_extract_).

#### ABTS^•+^ scavenging assay

The assay was carried out by the method of Re and colleagues [13] with slight modifications. Firstly, phosphate buffered saline (PBS) solution (75 mM/L; pH 7.4) was prepared by dissolving 8.18 g NaCl, 0.27 g, KH_2_PO_4_, 3.58, Na_2_HPO_4_ × 12H_2_O and 0.15 g KCl in 1 L of distilled water. Stock ABTS^•+^ solution was prepared by mixing 50 mL ABTS (2 mM/L PBS) with 200 μL K_2_S_2_O_8_ (70 mM/L H_2_O) and keeping for 12–16 h at room temperature in the dark. Before each assay, stock ABTS^•+^ solution was diluted with PBS to obtain the working solution with absorbance of 0.80±0.03 at 734 nm. For analysis 25 μL of sample or MeOH (blank) were mixed with 1500 μL of working ABTS^•+^ solution and left in dark for 2 hours.

#### Ferric reducing antioxidant power (FRAP)

The FRAP assay was carried out by the method of Benzie and Strain [14] with some modifications. FRAP reagent was prepared by mixing a solution of 10 mM TPTZ (in 40 mM HCl), 20 mM FeCl_3_∙6H_2_O and acetate buffer (300 mM, pH 3.6) at 1:1:10 (v/v/v). For measurement 50 μL of sample or MeOH (blank) were mixed with 150 μL of distilled H_2_O and 1500 μL of freshly prepared FRAP reagent. After 2 h incubation in the dark, the decrease in absorbance was read at 593 nm. A series of Trolox solutions in the concentration ranges of 0–800 μmol/L MeOH were used for the calibration and the results were expressed in μM TE/g_extract_.

#### Oxygen radical absorbance capacity (ORAC)

ORAC assay was carried out by using fluorescein as a fluorescent probe [15]. For analysis 25 μL of sample or MeOH (blank) were mixed with 150 μL of fluorescein solution (14 μmol/L PBS) in a black clear-bottom 96-well opaque microplate. The mixture was preincubated for 15 min at 37°C and 25 μL of AAPH solution (240 mmol/L PBS) as a peroxyl radical generator immediately added using multichannel pipet. The fluorescence was recorded every 1 min at 485 excitation and 520 emission wavelengths during 150 min at 37°C using FLUOstar Omega reader (BMG Labtech, Offenburg, Germany). Trolox solutions (0–250 μmol/L PBS) were used for calibration. The final ORAC values were calculated by using a regression equation between the Trolox concentration and the net area under the curve (AUC) as follows: AUC = (1+f1/f0+f2/f0…fi/f0…), where f0 is the initial fluorescence reading at time 0 min and fi is fluorescence reading at time i, and expressed in μM TE/g_extract_.

### Identification and quantification of phenolic compounds by UPLC-MS analysis

Chromatographic separation of analytes was carried out on an Acquity UPLC (Waters, Milford, MA, USA) system equipped with a binary pump, autosampler, photodiode array (PDA) detector, column manager, data station running the Compass acquisition and data software. Compounds were separated on an Acquity BEH, C18 column (100 mm × 2.1 mm, 1.7 μm) maintained at 40°C. The eluent system consisted of solvents A (0.1% formic acid in ultra pure water) and B (100% acetonitrile) with a linear gradient programmed as follows: 0.0–14 min, 5% B; 15–17 min, 100% B; 18 min, 5% B. The flow rate was 0.4 mL/min, temperature of sample 12°C and sample injection volume 1 μL. The effluents from the PDA detector were introduced directly into the quadrupole-time of flight mass spectrometer (Q-TOF) equipped with an electrospray ionization source controlled by HyStar 3.2 SR2 software (Bruker Daltonic, Bremen, Germany). All MS data were recorded in ESI negative ionization mode in a range of 80–1200 m/z, the capillary voltage was maintained at +4000 V. Nitrogen was used as a nebulizer gas at 2.5 bar and drying gas at flow rate of 10 L/min. The peaks were identified by comparing their retention times and parent ions with external standards, references and commercial databases.

Selected phenolics were quantified using an Acquity UPLC^TM^ H-Class equipped with Xevo TQ-S tandem quadrupole mass spectrometer (Waters, Milford, MA) operating in negative electrospray ionization (ESI) mode, capillary voltage was set to 1500 V, cone voltage to 20 V, source offset to 50 V. Desolvation temperature was 450°C, desolvation gas flow 1000 L/h, cone gas flow 150 L/h and nebulizer gas flow was set to 7 L/h. Chromatographic separation was performed using the same column and solvents as described above with a programmed linear gradient: 0.0–7 min, 5% B; 8–9 min, 50,7% B; 10–11 min, 100% B; 12–20 min, 5% B. The flow rate was 0.4 mL/min and sample injection volume was 5 μL. MS detection was achieved in the single-ion-monitoring (SIM) mode. The m/z values and dwell times of components were set as follows: 169.1595 m/z and 0.1 s (gallic acid), 301.0957 m/z at 0.025 s (ellagic acid), 447.1595 m/z at 0.025 s (quercitrin), 609.2233 m/z at 0.025 s (rutin), 951.1957 m/z at 0.050 s (geraniin), 991.1000 m/z at 0.025 s (phyllanthusiin D), 1109.1000 m/z at 0.050 s (elaeocarpusin). MassLynx 4.1 software was used for instrument control and data collection. All samples were run in triplicates. The concentrations of phytochemicals were calculated from calibration curves prepared using 0.05–50 μg/mL concentrations of reference compounds: gallic acid (y = 34937x-16.54; R^2^ = 0.9937), ellagic acid (y = 6142.6x+16634; R^2^ = 0.9954), geraniin (y = 6158x+4127; R^2^ = 0.9986), rutin (y = 45440x+28682; R^2^ = 0.9953) and quercitrin (y = 65477x+19631; R^2^ = 0.9953). The results were expressed both in the dry weight of extracts (DWE) and in the dry weight of the whole plant material (DWP). For the determination of fragmentation patterns of some compounds, direct infusion was made to a Waters TQ-S by deploying collision induced dissociation (CID) using argon as a collision gas at 25 eV and a flow rate of 0.11 mL/min.

### Statistical analysis

Statistical analysis was performed using GraphPad Prism software (version 5.0; GraphPad software, La Jola, CA, USA). Results were subjected to analysis of variance (one-way ANOVA) and the differences between means were calculated using Tukey’s multiple comparison test. They were considered significant when P-values were below 0.05 (P < 0.05). All data were expressed as means±SD.

## Results and discussion

### Cytotoxic effect of *P*. *phillyreifolius* extracts

**Fig 1:**
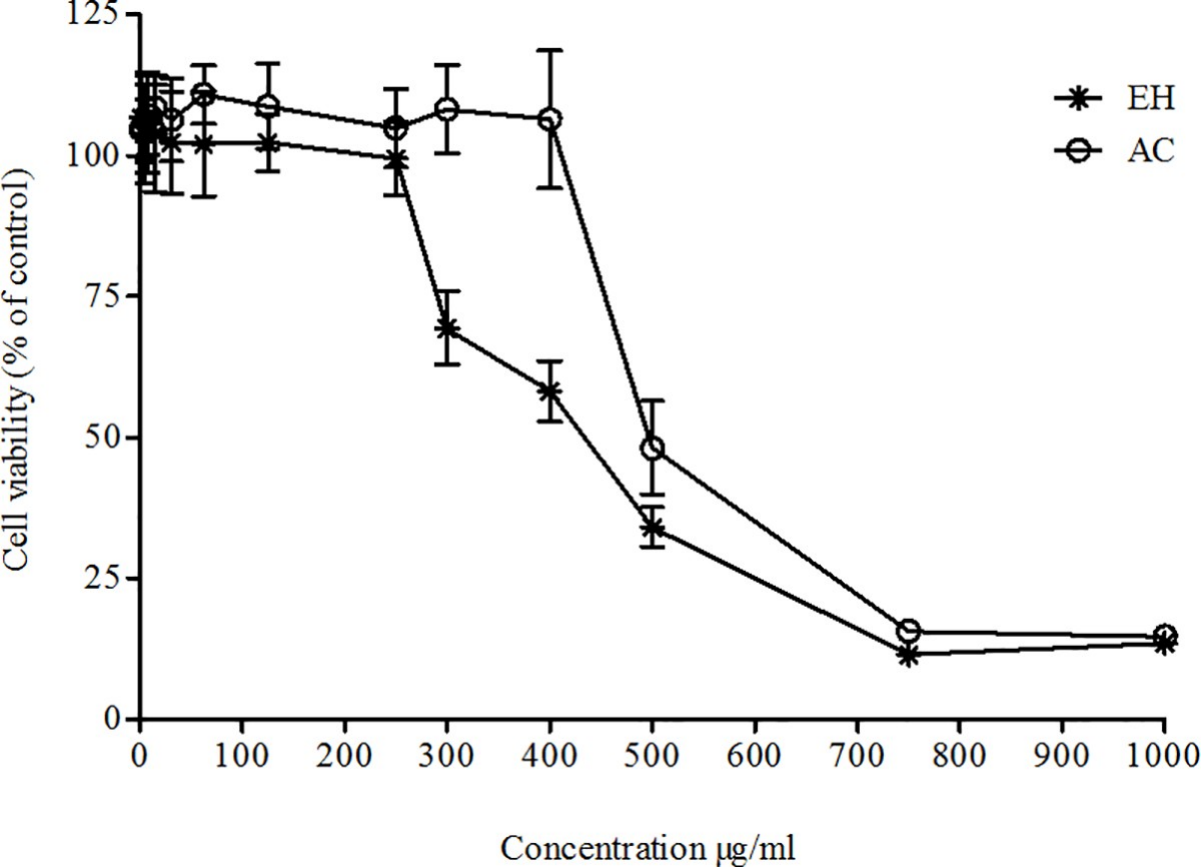
Effect of *P*. *phillyreifolius* extracts on HEK293 cells viability analysed by mitochondrial metabolic activity (MTT) assay. Cells were treated for 24 h with increased concentration of hydroethanolic (EH) or acetonic (AC) extracts of *P*. *phillyreifolius*. Data are represented as means ± standard deviations (n = 3).

The assessment of plant extracts for potential cytoxicity is considered as an important step in evaluating their suitability for further applications [16]. The cytotoxic effect of AC and EH extracts of *P*. *phillyreifolius* at 12.5–1000 μg/mL concentrations was investigated using HEK293 cell line by MTT cell viability assay, which relies on mitochondrial metabolic capacity of viable cells. Cell viability results after 24 h of incubation with *P*. *phillyreifolius* extracts are presented in Fig 1. It may be observed that cell viability decreased with the increase of extracts concentration; both extracts were toxic at higher than 500 μg/mL concentration. However, the cells exposed to lower extract concentrations (< 250 μg/mL) retained higher than 90% cell viability. This is in agreement with the previously reported results [17], showing that methanolic extract of the same genus plant, *P*. *acidus* was not cytotoxic to RAW264.7, U937 and HEK293 cells at the concentrations of up to 300 μg/mL. The concentrations of extracts responsible for a 50% reduction in cells viability (IC_50_) were 489±31.8 and 387±6.84 μg/mL for AC and EH extracts, respectively. The obtained results indicate on higher cytotoxic potential of EH extract than the AC one. Based on these results, ≤250 μg/mL concentrations of AC and EH extracts were chosen for treating cells in further experiments.

### Effect of *P*. *phillyreifolius* extracts on intracellular ROS level in HEK293 cells

The effect of preconditioning of HEK293 cells with EH and AC extracts of *P*. *phillyreifolius* on intracellular ROS level was determinated using the cell-based assay, in which the DCFH-DA fluorescent probe is used as an indicator of ROS and oxidative stress. The nonpolar and nonionic DCFH-DA probe passively diffuses into the cells and is hydrolyzed by intracellular esterases to form nonfluorescent 2′,7′-dichlorofluorescein (DCFH). In the presence of ROS, intracellular oxidases and oxidants DCFH is oxidized to fluorescent 2′,7′-dichlorofluorescein (DCF), which is trapped inside the cells [18]. When ROS production is not compensated by the cellular antioxidant defense system, oxidative stress occurs [19]. Bioactive compounds can quench this reaction and prevent the generation of DCF. This can be accomplished on the cell membrane surface or within the cell following uptake of the antioxidant compounds [6].

**Fig 2:**
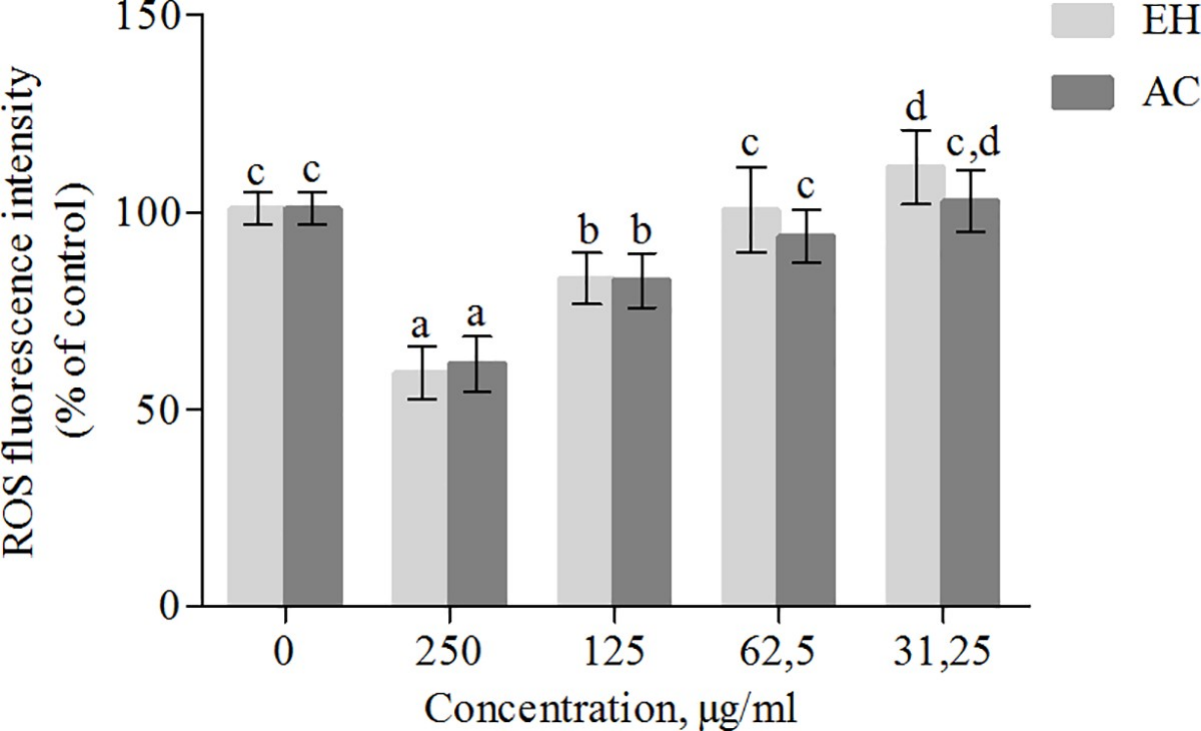
Effect of *P*. *phillyreifolius* extracts on basal level of ROS. HEK293 cells were treated with the noted concentrations of acetonic (AC) and hydroethanolic (EH) extracts for 24 h, then were washed and incubated with DCFH-DA (fluorescent probe as indicator of ROS) for 45 min. Data are represented as means ± standard deviations (n = 3) with one way ANOVA. The columns with different letters (a-d) differ significantly for Tukey’s test at p < 0.05.

**Fig 3:**
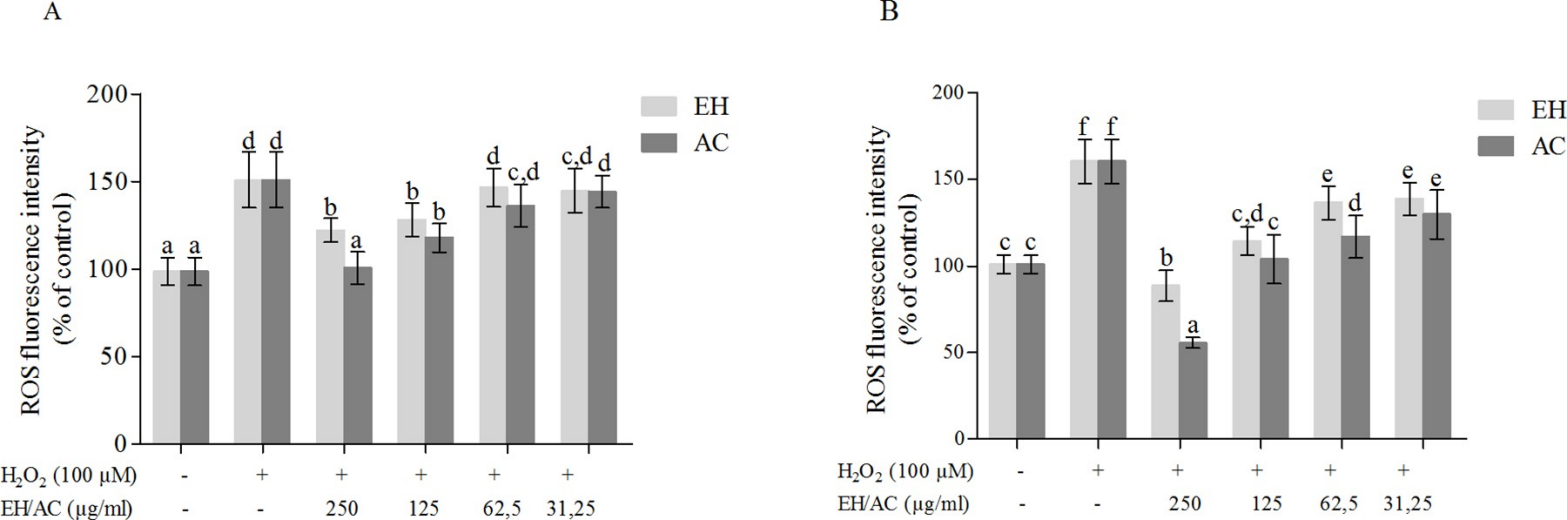
Effect of *P*. *phillyreifolius* extracts on ROS generation induced by H_2_O_2_. HEK293 cells were treated with extracts for A–3 h, B–24 h, then were washed, incubated with DCFH-DA for 45 min. Then supernatant was removed and cells were incubated for 1 h with H_2_O_2_ (100 μmol/L). Data are represented as means ± standard deviations (n = 3) with one way ANOVA. The columns with different letters (a-e) differ significantly for Tukey’s test at p < 0.05.

In order to evaluate the protective action of *P*. *phillyreifolius* extracts against oxidative stress, firstly the effect of extracts on the intracellular ROS content was determined in HEK293 cells without inducing exogenous oxidative stress. The cells were preconditioned with various concentration *P*. *phillyreifolius* extracts for 24 h. As shown in Fig 2. *P*. *phillyreifolius* extracts decreased the basal level of ROS in a dose dependent manner. Similar effect was observed in previous study undertaken by Septembre-Malaterre and colleagues [20], where passion fruit, litchi and American mango extracts decreased basal level of ROS in 3T3-L1 murine preadipose cells. To model oxidation stress in cells, the H_2_O_2_ was chosen as an intracellular oxidizing agent. The optimal concentration of H_2_O_2_ to induce oxidation was selected 100 μM since this concentration did not show any significant decrease in cell viability and at this concentration DCF had good fluorescence intensity in comparison with control. Two different preconditioning times with extracts (3 and 24 hours) were chosen to evaluate the antioxidant activity in H_2_O_2_-stimulated HEK293 cells. As showed in Fig 3, while H_2_O_2_ increased ROS production (from 98.7±5.74 to 150.9±7.89% of control), cells preconditioning with non toxic concentrations of *P*. *phillyreifolius* extracts significantly inhibited ROS generation in a dose dependent manner. Although cells preconditioning with extracts for 3 hours was sufficient time to inhibit ROS production, the treatment with 250 and 125 μg/mL by both extracts for 24 hours completely counteracted the H_2_O_2_ induced oxidative stress. In addition, AC extract showed better intracellular antioxidant activity that the EH one. The possible mechanism of ROS generation decreasing effect in cells pretreated with *P*. *phillyreifolius* extracts might be associated with the direct free radical scavenging activity or an indirect protection from oxidative stress, e.g. by activating endogenous defense systems.

### Expression of antioxidant enzymes in HEK293 cells

**Fig 4:**
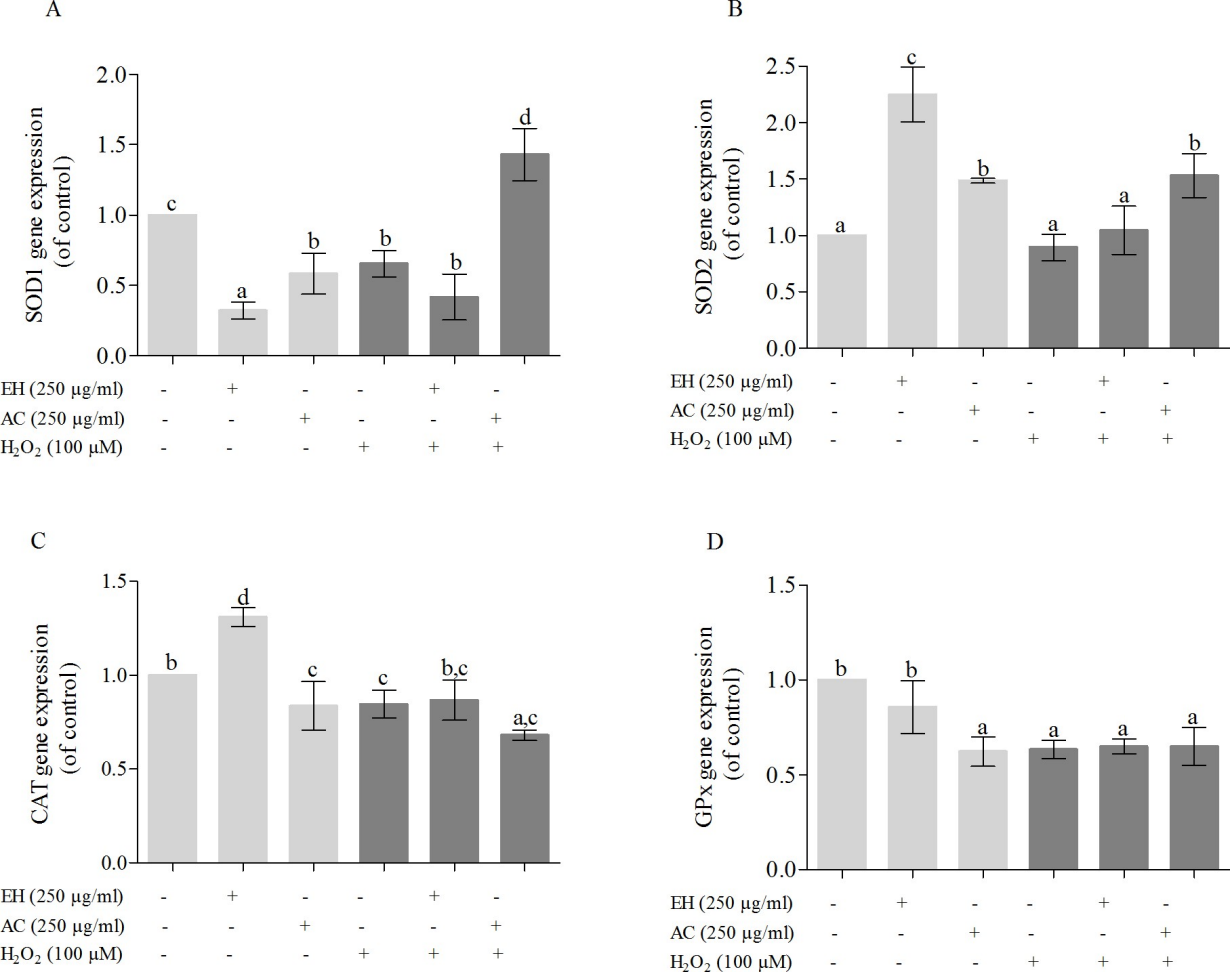
**Effect of *P*. *phillyreifolius* extracts on A–SOD1, B–SOD2, C–CAT, D–GPx enzymes gene expression in unstressed and H_2_O_2_-stimulated HEK293.** Cells were treated with extracts for 24 h, then were washed and incubated for 1 h with H_2_O_2_ (100 μmol/L). Data are represented as means ± standard deviations (n = 3) with one way ANOVA. The columns with different letters (a-d) differ significantly for Tukey’s test at p < 0.05.

There is an increasing evidence showing that some antioxidants act as cellular signaling messengers regulating the level of antioxidant compounds and enzymes [21]. So, in order to investigate whether ROS decreasing effect of plant extracts are mediated by an increase in antioxidant enzymes level, the preconditioning effect (24 h) of *P*. *phillyreifolius* extracts (250 μg/mL) on gene expression of antioxidant enzymes (SOD1, SOD2, CAT and GPx) was tested in H_2_O_2_-stimulated HEK293 cells (Fig 4). The gene expression of antioxidant enzymes in preconditioning HEK293 cells with AC and EH extracts was regulated differently. The gene expression of SOD2 and CAT was upregulated and SOD1 downregulated by preincubation cells with EH extract, meanwhile gene expression of SOD2 enzyme increased and SOD1, CAT, GPx enzymes decreased in cells pretreated with AC extract. Corroborating to our results it was reported that some doses of tested quercetin (well known antioxidant compound) were able to up- or down-regulate gene expression of the main antioxidant enzymes, though the acting mechanism was unknown [22]. A small but significant decrease in gene expression of SOD1, CAT and GPx was obtained in H_2_O_2_-stimulated HEK293 cells. Similar findings were reported in another study [5], showing that human hepatoma HepG2 cells treated with 200 μM of H_2_O_2_ significantly reduced the expresions of CuZnSOD, MnSOD, CAT and GPx enzymes, when compared with the untreated control. However, cells preincubation with *P*. *phillyreifolius* extracts before exposure to H_2_O_2_ didn‘t effect the antioxidant enzymes level, except the preconditioning with AC extract markedly increased the gene expression of SOD1 and SOD2 enzymes. Previous studies have shown different effect of polyphenol-rich extracts on antioxidant gene expression in H_2_O_2_ stimulated cells. For instance, it was shown that increase in ROS production induced by H_2_O_2_ in preadipocytes cells was related with decrease in SOD gene expression, which was counteracted by *A*. *borbonica* and *D*. *apetalum* extracts, though *G*. *mauritania* decreased the ROS generation without effect on SOD gene expression [19]. In adition, the absence of effect on catalase gene expression was also demonstrated in this study. Wang and colleagues [7] also reported, that antioxidant activities of *Acanthopanax senticosus* Harms aqueous extracts may be related to the upregulation of gene expression and activity of CuZnSOD, MnSOD, CAT, GPx1 enzymes. From the results obtained, it can be assumed that intracellular antioxidant activity of *P*. *phillyreifolius* extracts against H_2_O_2_ induced ROS production was more related with other acting mechanism compared to regulation of the antioxidant enzymes level.

### Total phenolic compounds and extracellular antioxidant activity

Taking into account previous studies showing that various antioxidants may exert protective effects against cellular damage by reacting with radicals and reducing oxidative stress [23], antioxidant properties of *P*. *phillyreifolius* extracts were also evaluated using extracellular *in vitro* assays. A single antioxidant assay cannot reflect all aspects of activities of natural products, because the antioxidant activity of plants may be attributed to a number of different mechanisms, including single electron transfer (SET) and hydrogen atom transfer (HAT). Many common antioxidant assays, including TPC, DPPH^•^ and ABTS^•+^ scavenging capacity and FRAP, are based on SET mechanism, in which electrons are transfered to reduce target compounds. Meanwhile ORAC assay is based on HAT mechanism and quantify hydrogen atom donating capacity [24, 25]. All these methods were used for assessing the extracellular antioxidant activity of *P*. *phillyreifolius* extracts.

The TPC of EH and AC extracts was 454±13.5 and 449±20.0 mg GAE/g_extract_, respectively_._ It may be observed that both extracts were abundant in phenolic compounds, however the difference was not significant (p<0.05). The TPC values measured for *P*. *phillyreifolius* were higher than previously reported for *P*. *debilis*, *P*. *amarus* and *P*. *urinaria* (21.3±3.6 to 205.3±21.3 mg GAE/g dry extract) [26]. Both investigated extracts demonstrated strong antioxidant activity in all assays; however, sslight differences may be observed. Antioxidant capacity of AC extract was higher than that of EH extract in DPPH^•^ (3395±121 and 3319±79 μM TE/g_extract_, respectively) and ABTS^•+^ scavenging reactions (7655±444 and 6887±138 μM TE/g_extract_), although in DPPH^•^ assay the difference was not significant (p<0.05). Antioxidant capacity of AC extract was also higher than EH extract in FRAP (6061±212 and 5567±185 μM TE/g_extract_, respectively) and ORAC assays (3443±113 and 3205±128 μM TE/g_extract_, respectively). These findings indicate that lower polarity AC extract possessed stronger antioxidant capacity in various assays than the polar EH extract. It should be noted that AC extract displayed better intracellular antioxidant activity too. These findings clearly indicate that extraction solvents should be selected individually for each plant source; for instance, numerous previously performed studies showed that high polarity methanolic extracts were the most effective in terms of recovery of polyphenols and antioxidant potential of the extracts obtained [27, 28].

### Characterization of phytochemicals by chromatography-mass spectrometry

**Fig 5:**
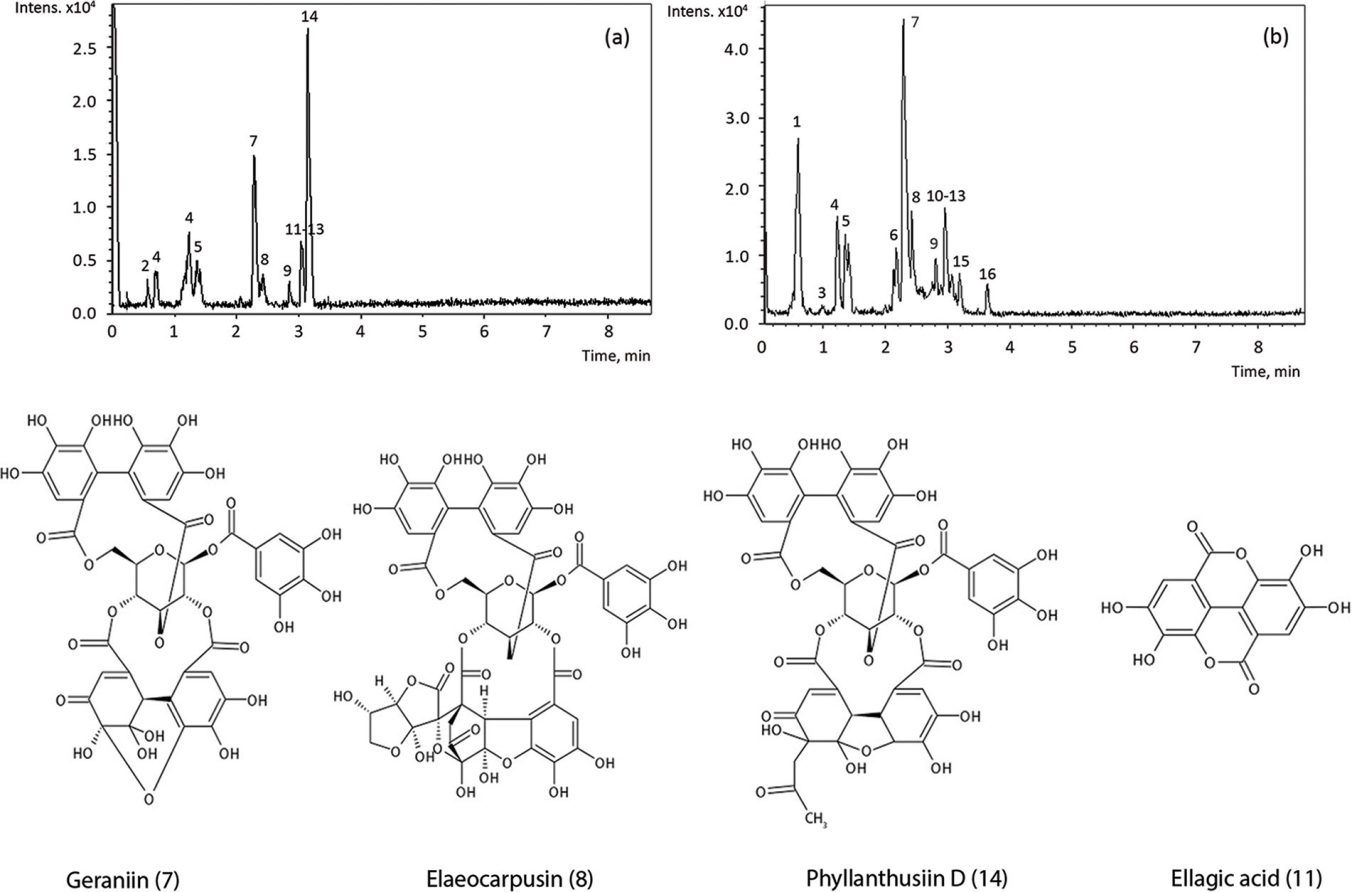
**Chromatograms of AC (a) and EH (b) extracts obtained by UPLC-Q-TOF and chemical structures of the main compounds, quantified in *P*. *phillyreifolius***: geraniin, elaeocarpusin, phyllanthusiin D and ellagic acid.

The representative chromatograms of extracts and the structures of the main quantified in *P*. *phillyreifolius* compounds are shown in Fig 5. Some of them were further quantified by UPLC-TQ-S using external standards and integrated peak areas. The concentrations of phenolics are expressed both in mg/g extract DWE and mg/g dry material (DWP) (Table 1).

**Table 1:** Chemical profile of *P*. *phillyreipholius* extracts analysed by UPLC-MS.

Peak No.	Compound	Molecular formula	RT (UPLC)	m/z [M-H]^-^ [M– 2H]^2–^	MS Fragments	AC		EH	
						mg/g DWE	mg/g DWP	mg/g DWE	mg/g DWP
1	Mucic acid lactone [2]*^b^	C_6_H_8_O_7_	0.50	191.0195	-	-	-	-	-
2.	Fructose*^c^	C_6_H_12_O_6_	0.55	179.0559	-	-	-	-	-
3.	Gallic acid^a^	C_7_H_6_O_5_	1.00	169.0141	-	0.78±0.06	0.09	0.52±0.03	0.18
4.	Unknown	C_15_H_20_O_10_	0.75; 1.25	359.0983	-	-	-	-	-
5.	Catalpol, antirrhinoside*^c^	C_15_H_22_O_10_	1.45	361.1139	-	-	-	-	-
6.	Phyllanthurinolactone*^c^	C_14_H_18_O_8_	2.15	313.0935	-	-	-	-	-
7.	Geraniin^a^	C_41_H_28_O_27_	2.20	951.0740 475.033	-	288±19.5	33.2	327±7.66	110
8.	Elaeocarpusin**^c^	C_47_H_34_O_32_	2.55	1109.0943 554.0445	1048.48, 972.56, 300.91 [EA-H]^-^	27.0±1.15	3.11	19.4±0.93	6.54
9.	Trigalloyl-HHDP-glucose*	C_41_H_28_O_27_	2.85	951.0742	-	-	-	-	-
10.	Unknown	C_42_H_32_O_27_	3.00	965.0889	-	-	-	-	-
11.	Ellagic acid^a^	C_14_H_6_O_8_	3.05	300.9984	-	51.5±1.15	5.94	40.11±0.09	13.49
12.	Rutin^a^	C_27_H_30_O_16_	3.10	609.1454	-	1.63±0.06	0.19	3.68±0.05	1.24
13.	Unknown	C_20_H_34_O_10_	3.10	433.2074	-	-	-	-	-
14.	Phyllanthusiin D**^c^	C_44_H_32_O_27_	3.15	991.1062 495.0490	990.61, 300.91 [EA-H]^-^	157±6.86	18.1	nd	nd
15.	Quercetin-3-Glucuronide*^c^	C_21_H_18_O_8_	3.25	477.0667	477.07, 301.04 [EA-H]	-	-	-	-
16.	Quercitrin^a^	C_21_H_20_O_11_	3.55	477.0924	-	0.24±0.01	0.03	1.04±0.01	0.35

^a^Confirmed by a standard

^b^confirmed by a reference

^c^confirmed by parent ion mass using free chemical databases (Chemspider)

*tentatively identified

**expressed in geraniin equivalents

nd: not detected. The values are represented as means ± standard deviations (n = 3), DWE–dry weight of extract; DWP–dry weight of initial plant.

Chemical composition of *P*. *phillyreifolius* and other, previously characterized species of the same genus [29], indicates that the plants contain considerable amounts of hydrolysable tannins, mainly ellagitannins, which are characterised as polyphenolic compounds with various numbers of hexahydroxydiphenoyl (HHDP) units attached to a sugar moiety [30]. It may be observed that ellagitannin geraniin with m/z = 991.1062 corresponding to molecular ion of C_41_H_27_O_27_ is quantitatively a major constituent of *P*. *phillyreifolius*; its structure was confirmed by comparing retention time with a commercial standard. Its concentration in the extracts was from 226 (ACp) to 327 (ETs) mg/g DWE, while the recovered amount varied from 13.2 (ACp) to 110 (ETs) mg/g DWP. In Japan, due to the presence of high amount of geraniin, *G*. *thunbergi* is certified as an official antidiarrheal drug [31], which supports the use of *P*. *phillyreifolius* in traditional medicine to this purpose. Since its discovery, geraniin has been credited with a range of bioactive properties that foster further evaluation of its potential as a promising active ingredient for pharmaceuticals, nutraceuticals and cosmetics [32]. Other two high molecular weight compounds of *P*. *phillyreifolius* showed characteristic to ellagitannins chromatographic and spectral properties such as a loss of hexahydroxydiphenyl unit (HHDP; 302 amu) and tendency to form double charged ions [33]. In acetonic extracts the compound with m/z = 991.1062 corresponding to molecular ion of C_44_H_31_O_27_ was assigned to phyllanthusiin D, which was previously isolated from aqueous acetonic extract of *P*. *amarus* [34] and is regarded as an artifact condensate of geraniin with acetone, produced during the extraction [35]. Another condensation product derived from geraniin and ascorbic acid with m/z = 1109.0943 corresponding C_47_H_33_O_32_ ion was also tentatively assigned to elaeocarpusin in the all extracts. This compound was previously reported in some *Acer*, *Rhus*, *Elaeocarpus* and *Cercidiphyllum* species [36]. Due to a high structural similarity to geraniin and difficulties in the availability of phyllanthusiin D and elaeocarpusin reference standards, both compounds were quantified using the calibration curve of geraniin and their amounts were expressed in geraniin equivalents. Phyllanthusiin D concentration among acetonic extracts varied from 35.6 (ACp) to 178 (ACc) mg/g DWE, while its recovery was from 2.08 (ACp) to 18.1 (ACs) mg/g DWP; the amounts of elaeocarpusin in extracts ranged from 14.2 (ETp) to 30.3 (ACp) mg/g DWE, its recovery was from 1.77(ACp) to 6.54 (ETs) mg/g DWP.

Flavonoids and phenolic acids were also detected in all *P*. *phillyreifolius* extracts. Gallic and ellagic acids were positively identified according to their chromatographic retention time and mass spectra by comparing with commercial standards. The concentration of ellagic acid in the exracts was from 31.5 (ETp) to 56.6 (ACc) mg/g DWE, while its recovery was from 2.25 (ACp) to 15.0 (ETc) mg/g DWP. The respective values for gallic acid were less than 1 mg/g. It is believed that ellagic acid in its free form does not naturally present in the plants; it forms when the HHDP group is cleaved from the tannin molecule and spontaneously rearranges; therefore, it is found in many fruits, nut galls and plant extracts in the form of ellagitannins [37]. Low concentrations of rutin and quercitrin were determined in the extracts using reference compounds. The compound with m/z = 477.0667 corresponding to molecular ion of C_21_H_17_O_8_ and characteristic fragment ion of m/z = 301 corresponding to the quercetin was tentatively identified as quercetin-3-glucuronide. This is in agreement with other studies, which reported quercetin-3-glucuronide in selected *Phyllanthus* species [29]. The identified phenolic acids and flavonoids were previously reported in other plants of the genus *Phyllanthus* [29]. It may be observed that chemical composition of acetonic and hydroethanolic extracts was quite similar; however, higher amount of the quantified constituents was found in EH extract than in AC extract which confirms that 70% ethanol extracted more phenolics than acetone.

High antioxidant potential of geraniin as a major compound might be responsible for the strong antioxidative activity of extracts. This is in agreement with the study of Wang and colleagues [7], who demonstrated, that geraniin attenuated H_2_O_2_ induced ROS production in a dose dependent manner as compared with the H_2_O_2_ treatment alone in human hepatocarcinoma cell line HepG2. Relatively high concentration of ellagic acid could also contribute to high antioxidant activity of extracts. It should be noted that Agbor et al. (2014), based on the *in vivo* studies with mice, concluded that antioxidant potential and the presence of various phytochemical constituents in the *Justicia hypocrateriformis* water extract might be antidiarrheal activity factors [38].

## Conclusion

This study showed that preconditioning of HEK293 cells with *P*. *phillyreifolius* extracts strongly inhibited the generation of ROS induced by H_2_O_2_, thus protecting the cells against oxidative stress. The results of the *in vitro* antioxidant activity assays were in a good agreement with antioxidant activity in cells; acetonic extract had better antioxidant effect than hydroethanolic one. Gene expression of antioxidant enzymes was also affected by the pretreatment with *P*. *phillyreifollius* extracts; however, at the applied experimental conditions, the lack of relationship between the changes in gene expression indicates that ROS production decreasing effect of extracts was more related to their capacity to scavenge free radicals and with their reducing power, than regulation of the antioxidant enzymes level. The other mechanisms could also be involved. According to cytotoxicity assay, both investigated extracts demostrated low level of cytotoxicity towards HEK293 cell line, reducing cell viability to 50% at the concentrations of 489±31.8 and 387±6.84 μg/mL for AC and EH extracts, respectively. Seven phenolic constituents were quantified in extracts of *P*. *phillyreifollius*, geraniin being the major quantitatively constituent, followed by phyllanthusiin D, ellagic acid, elaeocarpusin, rutin, quercitrin and gallic acid. In general, the results obtained suggest that *P*. *phillyreifolius* is a promising source of antioxidants and provide preliminary support for the traditional use of this plant in treating diarrhea. Moreover, they are in line with a current tendency of searching and evaluating natural therapeutic agents, which might be based on plant phytochemicals and also considering the existing scientific evidence about adverse effects of various synthetic drugs [39]. Consequently, our findings may foster further studies of *P*. *phillyreifollius* bioactivities and health effects.

## Supporting information

S1 Table List of primers used for RT-qPCR. (PDF)
