# Supplementary material for: Protective effects of Phyllanthus phillyreifolius extracts against hydrogen peroxide induced oxidative stress in HEK293 cells
Source: PLoS One. 2018 Nov 16;13(11):e0207672. doi: 10.1371/journal.pone.0207672 (PMC6239330; doi:10.1371/journal.pone.0207672)
Supplement: S1 Table — (PDF) [file pone.0207672.s001.pdf]

Supplementary data

S1 Table. List of primers used for RT-qPCR

Gene	Sense primer (5‘-3‘)	Antisense primer (3‘-5‘)
SOD-1	ACCAGTGTGCGGCCAATGATG	GCTGTACCAGTGCAGGTCCTCA
SOD-2	AAGCTGACGGCTGCATCTGTTG	CGTGCTCCCACACATCAATCCC
GPx	AGTTCGGACATCAGGAGAA	AGGGCTTCTATATCGGGTTC
CAT	AGTCTCGCCGCATCTTCAACAG	TCTGGGACTTCTGGAGCCTACG
GAPDH	GGGAGCCAAAAGGGTCATCA	TGATGGCATGGACTGTGGTC
